# Exosomes Derived from *Akt*‐Modified Human Umbilical Cord Mesenchymal Stem Cells Improve Cardiac Regeneration and Promote Angiogenesis via Activating Platelet‐Derived Growth Factor D

**DOI:** 10.5966/sctm.2016-0038

**Published:** 2016-09-22

**Authors:** Jie Ma, Yuanyuan Zhao, Li Sun, Xiaochun Sun, Xiaosu Zhao, Xiaoxian Sun, Hui Qian, Wenrong Xu, Wei Zhu

**Affiliations:** ^1^ School of Medicine, Jiangsu University, Zhenjiang, Jiangsu, People's Republic of China; ^2^ Weifang People's Hospital, Weifang, Shandong, People's Republic of China; ^3^ The Affiliated Hospital, Jiangsu University, Zhenjiang, Jiangsu, People's Republic of China

**Keywords:** Akt, Exosomes, Mesenchymal stem cells, Cardioprotection, Angiogenesis, Platelet‐derived growth factor D

## Abstract

We have previously demonstrated the cardioprotective effects of exosomes derived from mesenchymal stem cells (MSCs). It is well known that the activation of Akt is involved in stem cell‐induced cardioprotection. In the present study, we investigated whether exosomes released from *Akt*‐overexpressing MSCs showed a beneficial effect on cardioprotection and angiogenesis. MSCs were collected from human umbilical cord (hucMSCs), and *Akt* was transfected into hucMSCs (Akt‐hucMSCs) by using an adenovirus transfection system. Exosomes were isolated from control hucMSCs (Exo) and Akt‐hucMSCs (Akt‐Exo). An acute myocardial infarction model was created by ligation of the left anterior decedent coronary artery (LAD) in rats. Various source exosomes (400 µg of protein) were infused via the tail vein immediately after LAD ligation. The cardiac function was evaluated by using echocardiography after different treatments for 1 and 5 weeks, respectively. Endothelial cell proliferation, migration, and tube‐like structure formation, as well as chick allantoic membrane assay, were used to evaluate the angiogenetic effects of Akt‐Exo. The results indicated that cardiac function was significantly improved in the animals treated with Akt‐Exo. In addition, Akt‐Exo significantly accelerated endothelial cell proliferation and migration, tube‐like structure formation in vitro, and blood vessel formation in vivo. The expression of platelet‐derived growth factor D (PDGF‐D) was significantly upregulated in Akt‐Exo. However, the angiogenesis was abrogated in endothelial cells treated with the exosomes obtained from MSCs transfected with PDGF‐D‐siRNA. Our studies suggest that exosomes obtained from *Akt*‐modified hucMSCs are more effective in myocardial infarction therapy through promoting angiogenesis. PDGF‐D plays an important role in Akt‐Exo‐mediated angiogenesis. Stem Cells Translational Medicine *2017;6:51–59*

**Significance Statement**

Cardioprotective effects of exosomes derived from mesenchymal stem cells (MSCs) have been demonstrated in previous studies. It is unknown whether exosomes released from *Akt* gene‐modified MSCs have a better effect on regenerating ischemic myocardium. This study suggests that exosomes released from *Akt* gene‐modified human umbilical cord MSCs are more effective in improving cardiac function through promoting angiogenesis, which may provide a basis for developing a new therapeutic strategy for acute myocardial infarction.

## Introduction

Acute myocardial infarction (AMI), a human disease with a high mortality rate, has attracted widespread attention. It has been reported that stem cell therapy can reduce apoptosis and fibrosis, improve ventricular remodeling, and increase blood vessel density in ischemic myocardial injury 1 2 3 4. Paracrine effect was known as a primary mechanism for stem cell‐based therapies 5, 6. A large number of growth factors, chemokines, and cytokines are involved in repairing the ischemic myocardium 7 8 9 10 11. Exosomes secreted from stem cells carry many bioactive molecules, which not only prevent cell apoptosis and promote cell proliferation, but also improve neovascularization 9 10 11 12. It is well known that Akt (protein kinase B), a crossing in a series of pivotal signaling pathways, plays an important role in promoting cell proliferation and inhibiting cell apoptosis. The activation of Akt is involved in stem cell‐induced cardioprotection 13. Moreover, it has been reported that *Akt*‐modified mesenchymal stem cells (MSCs) mediated cardiac protection and functional improvement through the paracrine effect 14, 15.

Human umbilical cord‐derived mesenchymal stem cells (hucMSCs) are in the foreground as a preferable application for treating diseases because of their lower immunogenicity 16, 17, higher proliferation factors, and more amenable transfection efficiency 18, 19. It has been reported that exosomes derived from hucMSCs relieved acute myocardial ischemic injury 11. Furthermore, it has been reported that *Akt*‐modified MSCs mediated cardiac protection 20. As compared with MSCs, exosomes are similar to their parents. However, they are also different from their parent MSCs because of many advantages, including long‐term storage stability, ease of being internalized into recipient cells, very low immune rejection, convenient administration, etc. It remains unknown whether exosomes derived from *Akt*‐modified hucMSCs (Akt‐Exo) showed a better effect on repairing ischemic myocardium. In this study, we investigated the effect of Akt‐Exo on acute myocardial ischemia. Akt‐Exo were intravenously administrated after ligation of the left anterior descending (LAD) coronary artery in rats. Multiple techniques were used to detect exosome‐mediated angiogenesis. The results indicated that Akt‐Exo significantly improved cardiac function and promoted blood vessel formation, which was abolished in exosomes obtained from MSCs transfected with platelet‐derived growth factor D (PDGF‐D) siRNA. Our studies suggest that PDGF‐D/platelet‐derived growth factor receptor (PDGFR) may play an important role in Akt‐Exo‐mediated myocardial repair.

## Methods

### Cell Culture, *Akt* Transfection, and PDGF‐D siRNA Interference

HucMSCs were isolated and cultured according to the verified method 21. HucMSCs were cultured in low‐glucose Dulbecco's modified Eagle's medium (L‐DMEM) with 10% fetal bovine serum (FBS) (Thermo Fisher Scientific Life Sciences, Oakwood Village, OH, https://www.thermofisher.com) in 5% CO_2_ at 37°C. Passage 3‐4 hucMSCs were transfected with *Akt* (Akt‐hucMSCs) and its control *GFP* (GFP‐hucMSCs) by using the adenovirus transfection system (Thermo Fisher). In brief, when hucMSCs reached 60% density, 2 × 10^8^ plaque‐forming unit per ml recombinant adenovirus (Ad‐Akt or Ad‐GFP) was added into the culture medium. After 48 hours, the transfection efficiency was identified. For PDGF‐D siRNA interference, Lipofectamine 2000 (Thermo Fisher) and PDGF‐D siRNA/control siRNA (RiboBio, Guangzhou, People's Republic of China, http://www.ribobio.com/siteen) were mixed according to the manufacturer's instructions. HucMSCs were suspended in serum‐free L‐DMEM for 20 minutes, and then the medium was changed to serum‐free L‐DMEM with PDGF‐D siRNA/control siRNA for 4 hours. Finally, the medium was replaced with L‐DMEM with 10% FBS. Human umbilical vein endothelial cells (EA.hy926) and rat myocardial cells H9C2(2‐1) were purchased from the Chinese Academy of Medical Sciences (Beijing, People's Republic of China, http://english.pumc.edu.cn) and cultured in high‐glucose DMEM (H‐DMEM) with 10% FBS in 5% CO_2_ at 37°C.

### Exosome Extraction and Characterization

Exosomes were extracted following the previous article 11. In brief, the 10% FBS L‐DMEM was replaced with 10% exosome‐free FBS L‐DMEM when cultured hucMSCs reached ∼80% to ∼90% density. The conditioned medium of hucMSCs was collected after 48 hours and centrifuged to remove dead cells and cell debris. Then, conditioned medium was concentrated by using a 100‐kDa molecular mass cutoff hollow fiber membrane (EMD Millipore, Billerica, MA, http://www.emdmillipore.com) at 1,000*g* for 30 minutes. The concentrated medium was loaded onto 5 ml of 30% sucrose/D_2_O cushions and ultracentrifuged at 100,000*g* for 2 hours (optimal‐90k, Beckman Coulter, Miami, FL, https://www.beckmancoulter.com). The bottom of the cushion containing the exosomes was collected and washed three times with phosphate‐buffered saline (PBS). Isolated exosomes were characterized by using a digital microscope LM10 system (Malvern Instruments, Malvern, UK, http://www.malvern.com). In brief, exosomes were suspended in PBS and then injected into the LM10 unit. The particle size, particle concentration, and video frame of exosomes were analyzed by using nanoparticle tracking analysis (NTA).

### AMI Model, Infusion of Exosomes and Echocardiography

Animal guidelines and protocols were approved by the Animal Experimental Center of Jiangsu University, People's Republic of China. The AMI model was created with healthy 220‐ to 250‐g Sprague‐Dawley rats following the reported method 11. In brief, rats were anesthetized by using 10% chloral hydrate (Sinopharm Chemical Reagent Company, Shanghai, People's Republic of China, www.reagent.com.cnd) (300 mg/kg) by intraperitoneal injection and mechanically ventilated (Alcott Biotech Company, Shanghai, People's Republic of China, http://www.alcbio.com). The animal's chest was opened, and the left anterior descending coronary artery was quickly and accurately ligated with a 6‐0 suture. Finally, the chest was closed by tightening the double purse suture. Animals were randomly divided into five groups—PBS, Exo, GFP‐Exo, Akt‐Exo, and a sham group, which was similar to the AMI model, except there was no LAD coronary artery ligation. There were nine rats in each group. Various exosomes (400 µg of protein) or PBS was infused via the tail vein immediately after LAD ligation. Cardiac function was evaluated by using echocardiography at the first and fifth weeks after exosome infusion. The left ventricular ejection fraction (LVEF), left ventricular shortening fraction (LVFS), left ventricular internal diameter at the end of diastole (LVID;d), and left ventricular internal diameter at the end of systole (LVID;s) were analyzed.

### Terminal Deoxynucleotidyl Transferase dUTP Nick End Labeling Assay

The number of apoptotic cells in the tissue sections was counted by the terminal deoxynucleotidyl transferase dUTP nick end labeling (TUNEL) assay using a cell apoptosis detection kit (Vazyme, Nanjing City, People's Republic of China, http://en.vazyme.com), according to the manufacturer's instructions. The randomly microscopic fields were recorded, and the TUNEL‐positive cells were calculated by using Image‐Pro Plus 6.0.

### Cell Proliferation Assay

H9C2(2‐1) cells, or EA.hy926, were suspended in 1,000 µl of H‐DMEM containing 10% FBS and plated into six‐well chambers. When cells proliferated into the log phase, the medium was replaced by H‐DMEM with 2% FBS, and cell numbers were recorded for hour 0. Then, various exosomes (100 µg/ml), including Exo, GFP‐Exo, and Akt‐Exo, were added into the culture medium, and cell numbers were counted after culture for 24 and 48 hours, respectively.

### Scratch‐Wound Assay

H9C2(2‐1) or EA.hy926 cells were seeded in six‐well plates. The cell monolayer was scratched with a sterile, 10‐μl pipette tip when cultured cells reached 90% density. Various exosomes (100 µg/ml) and PBS (equal volume to exosomes) were added into the medium. The marked areas were captured by using a microscope (SMZ‐168, Motic, Xiamen, People's Republic of China, http://www.motic.com) before and after different treatments for 12 hours.

### Tube‐Like Structure Formation Assay

EA.hy926 cells (2 × 10^4^ per well) were suspended in 100 μl of H‐DMEM containing exosomes (100 µg/ml) or PBS and plated under 50 μl of Matrigel (BD Biosciences) in 96‐well plates after being solidified at 37°C for 30 minutes. After being incubated in 5% CO_2_ at 37°C for 12 hours, three random fields were photographed under an inverted microscope, and the numbers of tubes were counted.

### Chick Allantoic Membrane Assay

Fertilized chicken eggs were incubated at 37°C with 70% humidity for 10 days. The vascularized areas of the chick allantoic membrane (CAM) were localized. A hole was drilled above the vascularized areas, penetrating through the outer shell membrane without injuring the CAM. Sterilized scraps of filter paper absorbed with 50 μl of L‐DMEM, Exo, GFP‐Exo, and Akt‐Exo were placed on top of the CAM in the corresponding groups. Then eggs were sealed and incubated for 5 days. The vascularized areas of CAM were washed with PBS and photographed.

### Western Blot

Western blot was performed by following the described procedure 11. In brief, exosomes, cells, or tissue were lysed in lysis buffer. The protein samples were electrophoresed by using 12% SDS gels, and then transferred onto polyvinylidene fluoride membranes (EMD Millipore). Subsequently, the membranes were incubated with the following monoclonal primary antibodies overnight: CD63 (1:300; SAB, Nanjing, People's Republic of China, http://www.sabbiotech.com), GFP (1:500; SAB), Akt (1: 500; SAB), vascular endothelial growth factor (VEGF) (1:500; Santa Cruz Biotechnology, Santa Cruz, CA, http://www.scbt.com), CD31 (1:500; Santa Cruz Biotechnology), transforming growth factor β (TGF‐β) (1:300; SAB), PDGFR‐β (1: 300; Santa Cruz Biotechnology), PDGF‐D (1:300; Santa Cruz Biotechnology), and glyceraldehyde‐3‐phosphate dehydrogenase (1:2,000; CWBIO, Beijing, People's Republic of China, http://www.cwbiotech.bioon.com.cn/). Then, horseradish peroxidase (HRP)‐conjugated anti‐rabbit or anti‐mouse IgG was applied as a secondary antibody (1:2,000; bioWORLD, Dublin, OH, https://www.bio-world.com) for 1 hour at room temperature. The target proteins were visualized by using the Luminata Crescendo Western HRP substrate (EMD Millipore) and analyzed by AlphaView SA software.

### Statistical Analysis

Statistical analysis was performed by using SPSS 16.0 software (IBM, Armonk, NY, http://www.ibm.com). Data were expressed as means ± SD ($\bar{x}\pm s$). For in vitro experiments, each group contained three samples, and each experiment repeated at least three times. Student's *t* test was used to compare two groups. One‐way analysis of variance (ANOVA) followed by a post hoc test was used to analyze the variance equal to or greater than three groups. Two‐way ANOVA was used to analyze the results of cell proliferation assay. A value of *p* < .05 was considered significant.

## Results

### Characterization of Exosomes

**Figure 1:**
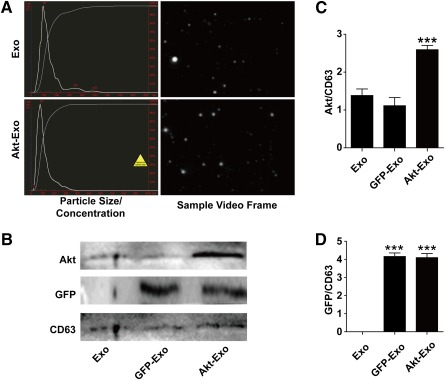
Characterization of exosomes. **(A):** The particle size, particle concentration, and video frame of Exo and Akt‐Exo were analyzed by nanoparticle tracking analysis. There were no significant differences between Exo and Akt‐Exo. **(B):** Representative images of Western blot in the protein expression of CD63, GFP, and Akt in exosomes. **(C, D):** Semiquantitative data of the expression of Akt **(C)** and GFP **(D)** to CD63. ∗∗∗, *p* < .001 versus Exo or GFP‐Exo. Abbreviations: Akt‐Exo, exosomes derived from human umbilical cord‐derived mesenchymal stem cells transfected with *Akt*; Exo, exosomes derived from untreated human umbilical cord‐derived mesenchymal stem cells; GFP‐Exo, exosomes derived from human umbilical cord‐derived mesenchymal stem cells transfected with *GFP*.

NTA was performed to characterize exosomes. The particle size and concentration did not show significant differences between Exo and Akt‐Exo (Fig. 1A). To confirm the transfection of *Akt*, the expression of Akt in exosomes was evaluated (Fig. 1B, 1C). The expression of Akt was significantly higher in Akt‐Exo than that in Exo and GFP‐Exo (Fig. 1C), although both GFP‐Exo and Akt‐Exo expressed GFP (Fig. 1D).

### Akt‐Exo Improved Cardiac Function

**Figure 2:**
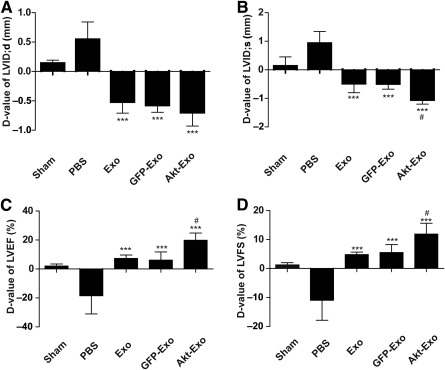
The cardiac function expressed as the difference between the first and fifth weeks following different treatments. **(A):** LVID;d. **(B):** LVID;s. **(C):** LVEF. **(D):** LVFS. ∗∗∗, *p* < .001 versus PBS group; #, *p* < .05 versus Exo group. Abbreviations: Akt‐Exo, exosomes derived from human umbilical cord‐derived mesenchymal stem cells transfected with *Akt*; D‐value, difference value; Exo, exosomes derived from untreated human umbilical cord‐derived mesenchymal stem cells; GFP‐Exo, exosomes derived from human umbilical cord‐derived mesenchymal stem cells transfected with *GFP*; LVEF, left ventricular ejection fraction; LVFS, left ventricular shortening fraction; LVID;d, left ventricular internal diameter at the end of diastole; LVID;s, left ventricular internal diameter at the end of systole; PBS, phosphate‐buffered saline.

LVEF, LVFS, LVID;d, and LVID;s were evaluated by using echocardiography at the first and fifth weeks after different treatments. The difference value (D‐value) of LVEF, LVFS, LVID;d, and LVID;s between the first and fifth weeks was used to compare the cardiac function. Compared with the PBS group, the D‐value of LVID;d and LVID;s in Exo‐treated, GFP‐Exo‐treated, and Akt‐Exo‐treated animals showed opposite trends (*p* < .001). Moreover, the D‐values of LVID;s were significantly different between Exo‐ and Akt‐Exo‐treated groups (*p* < .05) (Fig. 2A, 2B). The D‐value of LVEF and LVFS in Exo‐, GFP‐Exo‐, and Akt‐Exo‐treated animals were significantly increased compared with that in animals treated with PBS (*p* < .001). Moreover, the D‐value of LVEF and LVFS in animals treated with Akt‐Exo was significantly increased compared with those treated with Exo (*p* < .05) (Fig. 2C, 2D).

### Effect of Akt‐Exo on Myocardial Cell Apoptosis, Proliferation, and Migration

**Figure 3:**
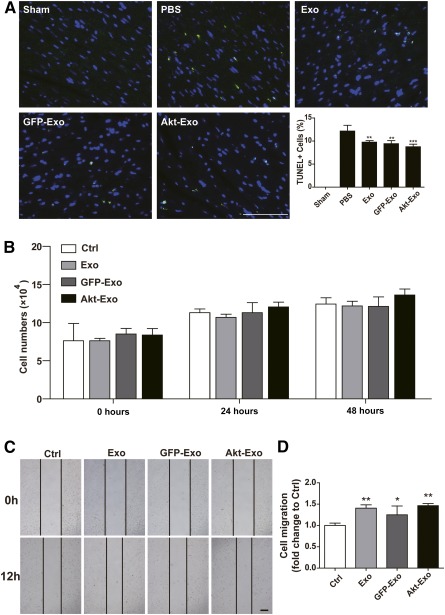
Effect of Akt‐Exo on myocardial cell apoptosis, proliferation, and migration. **(A):** Representative images of TUNEL staining of myocardium and the percentage of TUNEL positive cells. Scale bar = 100 μm. ∗∗, *p* < .01; ∗∗∗, *p* < .001 versus PBS group. **(B):** The numbers of H9C2(2‐1) cells following different treatments at 0, 24, and 48 hours. **(C):** Scratch‐wound assay of H9C2(2‐1) cells. Scale bar = 100 μm. **(D):** The distance that H9C2(2‐1) cells migrated. ∗, *p* < .05; ∗∗, *p* < .01 versus Ctrl group. Abbreviations: Akt‐Exo, exosomes derived from human umbilical cord‐derived mesenchymal stem cells transfected with *Akt*; Ctrl, control; D‐value, difference value; Exo, exosomes derived from untreated human umbilical cord‐derived mesenchymal stem cells; GFP‐Exo, exosomes derived from human umbilical cord‐derived mesenchymal stem cells transfected with *GFP*; PBS, phosphate‐buffered saline; TUNEL, terminal deoxynucleotidyl transferase dUTP nick end labeling.

To explore the antiapoptotic effect of Akt‐Exo on myocardial cells, TUNEL staining was processed in the myocardium after LAD ligation for 1 week. The number of TUNEL‐positive cells was significantly reduced in the animals treated with different exosomes compared with the animals treated with PBS. However, no significant difference could be seen among the animals treated with different exosomes (Fig. 3A).

The proliferation of H9C2(2‐1) was evaluated after various exosome treatments at 0, 24, and 48 hours. The results showed that H9C2(2‐1) proliferated very slowly, and no significant difference could be seen among the different treatments (Fig. 3B). The migration of H9C2(2‐1) was analyzed by using scratch‐wound assay. After 12 hours of culture, the migration distance in cells treated with Exo, GFP‐Exo, and Akt‐Exo was significantly increased compared with that in control, but no significant difference could be seen among the cells treated with different exosomes (Fig. 3C, 3D).

### Akt‐Exo Accelerated Endothelium Cell Proliferation, Migration, and Vessel Formation

**Figure 4:**
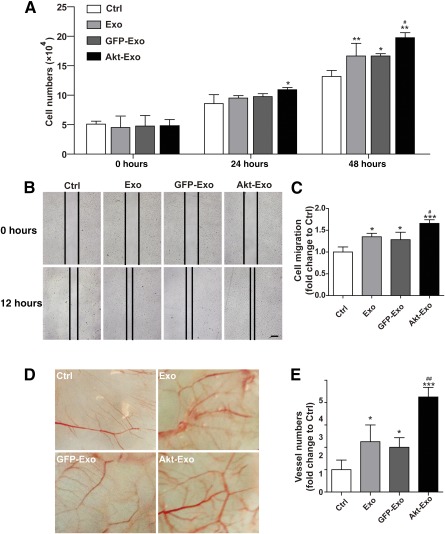
Akt‐Exo accelerated EA.hy926 cell proliferation and migration and promoted blood vessel formation in chick allantoic membrane. **(A):** The numbers of EA.hy926 cells in different treatments at 0, 24, and 48 hours. **(B, C):** Scratch‐wound assay to show EA.hy926 cell migration **(B)** and quantitative data **(C)**. Scale bar = 100 μm. **(D, E):** Representative blood vessel formation in chick allantoic membrane **(D)** and quantitative data of vessel numbers **(E)**. ∗, *p* < .05; ∗∗, *p* < .01; ∗∗∗, *p* < .001 versus Ctrl group; #, *p* < .05; ##, *p* < .01 versus Exo group. Abbreviations: Akt‐Exo, exosomes derived from human umbilical cord‐derived mesenchymal stem cells transfected with *Akt*; Ctrl, control; Exo, exosomes derived from untreated human umbilical cord‐derived mesenchymal stem cells; GFP‐Exo, exosomes derived from human umbilical cord‐derived mesenchymal stem cells transfected with *GFP*.

Neovascularization is one of the important therapeutic mechanisms in stem cell‐mediated ischemic myocardial regeneration. The effects of Akt‐Exo on endothelium cell proliferation, migration, and vessel formation assay were evaluated. The number of endothelial cells (EA.hy926) treated with different exosomes were counted at 0, 24, and 48 hours. The number of EA.hy926 cells treated with Akt‐Exo was significantly increased compared with that in the control group, although the cell number did not show a significant increase in the group treated with Exo or GFP‐Exo at 24 hours (Fig. 4A). Moreover, the cell number was further increased in the group treated with Akt‐Exo compared with other groups at 48 hours (Fig. 4A).

Endothelial cell migration was analyzed by using scratch‐wound assay. After 12 hours of culture, the migration distance was significantly greater in EA.hy 926 cells treated with Akt‐Exo compared with that treated with Exo or GFP‐Exo (Fig. 4B, 4C).

To investigate the effect of Akt‐Exo on blood vessel formation in vivo, the vascularized areas of CAM were photographed. After being incubated with exosomes for 5 days, the blood vessel number was significantly increased in CAM treated with exosomes compared with that incubated with L‐DMEM, and more blood vessels were found in CAM treated with Akt‐Exo (Fig. 4D, 4E).

### PDGF‐D/PDGFR is Involved in Akt‐Exo‐Mediated Myocardial Injury Repair

**Figure 5:**
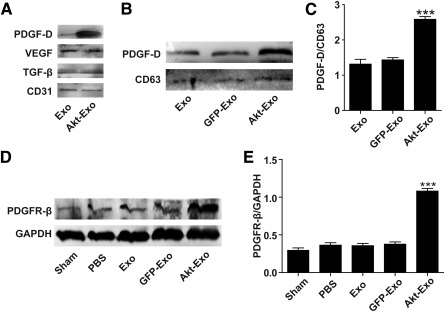
PDGF‐D/PDGFR is involved in Akt‐Exo‐mediated improvement of myocardial repair. **(A):** Representative images of Western blot on the expression of VEGF, CD31, PDGF‐D, and TGF‐β in exosomes. **(B, C):** Western blot of the expression of PDGF‐D **(B)** and semiquantitative ratio of PDGF‐D to CD63 **(C)**. **(D, E):** Representative images of Western blot of the expression of PDGFR‐β in myocardium **(D)** and semiquantitative data of PDGFR‐β to GAPDH **(E)**. ∗∗∗, *p* < .001 versus other groups. Abbreviations: Akt‐Exo, exosomes derived from human umbilical cord‐derived mesenchymal stem cells transfected with *Akt*; Exo, exosomes derived from untreated human umbilical cord‐derived mesenchymal stem cells; GAPDH, glyceraldehyde‐3‐phosphate dehydrogenase; GFP‐Exo, exosomes derived from human umbilical cord‐derived mesenchymal stem cells transfected with *GFP*; PBS, phosphate‐buffered saline; PDGF‐D, platelet‐derived growth factor D; PDGFR‐β, platelet‐derived growth factor receptor β; TGF‐β, transforming growth factor β; VEGF, vascular endothelial growth factor.

It is well known that exosomes deliver bioactive molecules and mediate the communication between cells. Some cytokines that regulate cell growth and angiogenesis, such as VEGF, CD31, PDGF‐D, and TGF‐β, were assayed in exosomes by using Western blot. The results showed that PDGF‐D expression level in Akt‐Exo was significant higher than that in other types of exosomes, although no significant difference could be seen in the expression of VEGF, TGF‐β, and CD31 between Akt‐Exo and Exo (Fig. 5A–5C). Furthermore, the expression level PDGFR‐β in the myocardium of animals treated with Akt‐Exo was significantly increased compared with that obtained from rats treated with PBS and other exosomes (Fig. 5D, 5E).

**Figure 6:**
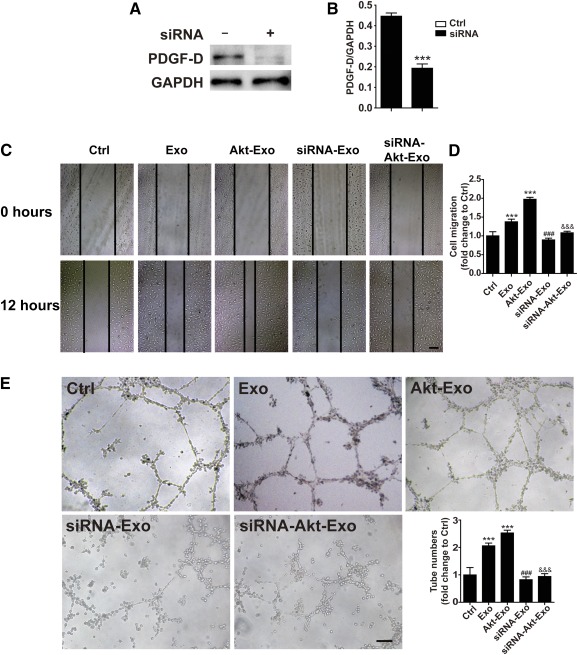
The role of PDGF‐D in Akt‐Exo‐mediated endothelial cell migration and tube formation. **(A, B):** Western blot of PDGF‐D in human umbilical cord‐derived mesenchymal stem cells transfected with or without PDGF‐D siRNA **(A)** and semiquantitative data **(B)**. ∗∗∗, *p* < .001 versus control cells. **(C, D):** Representative scratch‐wound photomicrographs of EA.hy926 in various groups **(C)** and quantitative data **(D)**. Scale bar = 100 μm. **(E):** Representative photomicrographs of tube‐like structure in various groups and quantification of tube numbers. Scale bar = 100 μm. ∗∗∗, *p* < .001 versus Ctrl group; ###, *p* < .001 versus Exo group; &&&, *p* < .001 versus Akt‐Exo group. Abbreviations: Akt‐Exo, exosomes derived from human umbilical cord‐derived mesenchymal stem cells transfected with *Akt*; Ctrl, control; Exo, exosomes derived from untreated human umbilical cord‐derived mesenchymal stem cells; GAPDH, glyceraldehyde‐3‐phosphate dehydrogenase; PDGF‐D, platelet‐derived growth factor D.

To investigate the effect of PDGF‐D on Akt‐Exo‐mediated angiogenesis, PDGF‐D‐siRNA was transfected into hucMSCs, and exosomes were collected from these cells. The results of the Western blot revealed that the PDGF‐D level exhibited was significantly lower in PDGF‐D‐siRNA‐transfected hucMSCs than in control hucMSCs (Fig. 6A, 6B). The migration of endothelial cells and tube formation assay were performed by using exosomes isolated from hucMSCs transfected with PDGF‐D‐siRNA (siRNA‐Akt‐Exo). The increased distance of EA.hy926 cell migration caused by Akt‐Exo was abrogated in EA.hy926 treated with siRNA‐Akt‐Exo (Fig. 6C, 6D). The tube‐like structure formation was also reduced in endothelial cells treated with siRNA‐Akt‐Exo compared with the cells treated with Akt‐Exo (Fig. 6E). These results indicated that PDGF‐D plays a critical role in Akt‐Exo‐mediated blood vessel formation.

## Discussion

It is well acknowledged that the paracrine effect plays an important role in stem cell‐mediated myocardial repair 6. Extracellular vesicles, including exosomes, secreted from stem cells reduce myocardial ischemia injury 9, 10. Our previous study also demonstrated that exosomes derived from hucMSCs protected myocardial cells from injury and improved cardiac function after myocardial infarction 11. In this study, exosomes were obtained from *Akt*‐transfected hucMSCs. The effects of Akt‐Exo on repairing ischemic myocardium and possible mechanisms were studied. Our results indicated that (a) Akt‐Exo showed a higher efficiency in improving cardiac function of AMI rats; (b) Akt‐Exo promoted endothelial cell proliferation, migration, and tube‐like structure formation in vitro and increased blood vessel formation in vivo; and (c) the beneficial effect of Akt‐Exo might be related to carrying a high concentration of PDGF‐D.

Echocardiography of experimental rats showed that there were significant differences between animals treated with Exo and Akt‐Exo in the D‐value of myocardial systolic‐related parameters, including LVEF, LVFS, and LVID;s. It is well known that cell loss is the main cause of acute or persistent ischemia‐induced myocardial damage. Antiapoptotic effect and promotion myocardial cell proliferation and migration effect have been taken into consideration in this study. However, Akt‐Exo do not have a significant beneficial effect in reducing cardiomyocyte injury compared with the effect of other exosomes. MSCs have been shown to perform their therapeutic roles by promoting angiogenesis 22 23 24. Therefore, neovascularization is considered to be one of the most important therapeutic mechanisms in Akt‐Exo‐mediated ischemic myocardial repair.

Neovascularization includes a set of processes, such as arteriogenesis, vasculogenesis, and angiogenesis, that are associated with migration and proliferation of endothelial cells. Therefore, cellular interventions for AMI have focused on promoting angiogenesis to stimulate the recovery of the microvasculature. A study by Bian et al. showed that extracellular vesicles derived from human bone marrow mesenchymal stem cells promoted angiogenesis in a rat myocardial infarction model 9. Our previous studies demonstrated that exosomes derived from hucMSCs enhanced angiogenesis through the Wnt4/b‐Catenin pathway 25. In this study, we further demonstrated that Akt‐Exo accelerated endothelium cell proliferation, migration, and vessel formation.

It is well known that therapeutic angiogenesis by delivery of vascular growth factors and cytokines is an attractive strategy for treating debilitating occlusive vascular diseases. Exosomes contain proteins (growth factors, enzymes, and cytokines), mRNAs, miRNAs, etc., which can be transferred to regulate the signaling pathway and function in recipient cells. Here, we assayed the expression of several growth factors and cytokines in Akt‐Exo. We found that PDGF‐D protein was significantly higher in Akt‐Exo compared with that in Exo, although no significant difference could be found in VEGF, CD31, and TGF‐β between the two types of exosomes. Moreover, PDGFR‐β protein expression level in myocardial cells treated with Akt‐Exo was also increased. Our study suggests that Akt‐Exo deliver more PDGF to recipient cells. It has been reported that PDGF inhibits pulmonary arterial endothelial cell damage in a dose‐dependent manner 26. In contrast, the inhibition of PDGFs causes increased apoptosis through an imbalance in the ratio of antiapoptotic to proapoptotic proteins 27. Moreover, PDGF promoted the maturation of the new blood branches 28, 29. It has been demonstrated that triiodothyronine‐induced cardiac sprouting angiogenesis in adult hypothyroid mice was associated with PDGF, PDGFR‐β, and downstream activation of Akt 30. In addition, it has been reported that the overexpression of PDGFR‐β can enhance the PDGF‐stimulated proliferation, migration, and angiogenesis of endothelial progenitor cells 31. To investigate the role of PDGF in Akt‐Exo‐mediated blood vessel formation, the exosomes were extracted from hucMSCs transfected with PDGF‐D‐siRNA. The promotion of tube formation induced by Akt‐Exo was markedly abolished in the cells treated with siRNA‐Akt‐Exo, supporting that PDGF‐D plays an important role in Akt‐Exo‐mediated angiogenesis.

## Conclusion

Our studies demonstrate that exosomes derived from *Akt* gene‐modified hucMSCs have a higher efficiency in repair of ischemic myocardium through promoting angiogenesis. Akt‐Exo delivers a high level of PDGF to endothelial cells and promotes their proliferation, migration, and formation of blood vessels. This study may provide a basis for developing a new therapeutic strategy to improve cardiac function for patients after AMI.

## Author Contributions

J.M., Y.Z.: data analysis and interpretation, collection and/or assembly of data; L.S. and Xiaoxian Sun: data analysis and interpretation; Xiaochun Sun and X.Z.: provision of study material or animals; H.Q.: conception and design, collection and/or assembly of data; W.X.: conception and design, financial support, administrative support; W.Z.: conception and design, manuscript writing, final approval of manuscript.

## Disclosure of Potential Conflicts of Interest

The authors indicated no potential conflicts of interest.
